# Association between ambient air pollutants and meteorological factors with SARS-CoV-2 transmission and mortality in India: an exploratory study

**DOI:** 10.1186/s12940-021-00804-0

**Published:** 2021-11-19

**Authors:** Sumit Aggarwal, Sivaraman Balaji, Tanvi Singh, Geetha R. Menon, Sandip Mandal, Jayaprakasam Madhumathi, Nupur Mahajan, Simran Kohli, Jasmine Kaur, Harpreet Singh, Kiran Rade, Samiran Panda

**Affiliations:** 1 Division of Epidemiology and Communicable Diseases, Indian Council of Medical Research-Headquarters, New Delhi, 110029 India; 2 Indian Council of Medical Research-National Institute of Medical Statistics, New Delhi, 110029 India; 3 World Health Organization, New Delhi, 110002 India

**Keywords:** COVID-19, SARS-CoV-2 transmission, Air pollutants, Meteorological, Parameters, Mortality, India

## Abstract

**Background:**

The Coronavirus disease 2019 (COVID-19) pandemic poses a serious public health concern worldwide. Certain regions of the globe were severely affected in terms of prevalence and mortality than other. Although the cause for this pattern is not clearly understood, lessons learned from previous epidemics and emerging evidences suggest the major role of ecological factors like ambient air pollutants (AAP) and meteorological parameters in increased COVID-19 incidence. The present study aimed to understand the impact of these factors on SARS-CoV-2 transmission and their associated mortality in major cities of India.

**Methods:**

This study used secondary AAP, meteorological and COVID-19 data from official websites for the period January-November 2020, which were divided into Pre-lockdown (January-March 2020), Phase I (April to June 2020) and Phase II (July to November 2020) in India. After comprehensive screening, five major cities that includes 48 CPCB monitoring stations collecting daily data of ambient temperature, particulate matter PM_2.5_ and _10_ were analysed. Spearman and Kendall’s rank correlation test was performed to understand the association between SARS-CoV-2 transmission and AAP and, meteorological variables. Similarly, case fatality rate (CFR) was determined to compute the correlation between AAP and COVID-19 related morality.

**Results:**

The level of air pollutants in major cities were significantly reduced during Phase I compared to Pre-lock down and increased upon Phase II in all the cities. During the Phase II in Delhi, the strong significant positive correlation was observed between the AAP and SARS-CoV-2 transmission. However, in Bengaluru, Hyderabad, Kolkata and Mumbai AAP levels were moderate and no correlation was noticed. The relation between AT and SARS-CoV-2 transmission was inconclusive as both positive and negative correlation observed. In addition, Delhi and Kolkata showed a positive association between long-term exposure to the AAP and COVID-19 CFR.

**Conclusion:**

Our findings support the hypothesis that the particulate matter upon exceeding the satisfactory level serves as an important cofactor in increasing the risk of SARS-CoV-2 transmission and related mortality. These findings would help public health experts to understand the SARS-CoV-2 transmission against ecological variables in India and provides supporting evidence to healthcare policymakers and government agencies for formulating strategies to combat the COVID-19.

**Supplementary Information:**

The online version contains supplementary material available at 10.1186/s12940-021-00804-0.

## Introduction

Air pollution and meteorological factors have been shown to influence the trends of respiratory disease outbreaks by altering host immunity and pathogen survival time [1]. These factors have also been reported to be the largest environmental determinants of disease and premature death in humans, including Severe Acute Respiratory Syndrome (SARS) and Middle East Respiratory Syndrome (MERS) [2, 3]. While Chronic Obstructive Pulmonary Diseases (COPD), respiratory illnesses and higher rates of hospital admission result from short-term exposure to such factors, long-term exposure to them has been associated with impaired lung function, asthma, lung cancer, heart attack, cardiovascular diseases and premature mortality [4].

The world is now facing a pandemic caused by the Severe Acute Respiratory Syndrome Coronavirus 2 (SARS-CoV-2) that was detected first in Wuhan, the capital city of Hubei province, China, in December 2019 [5]. Most nations were forced to declare complete lockdown to contain this viral transmission. Evidence from across the globe suggests that since the major route of SARS-CoV-2 transmission is through respiratory droplets of the infected people, there is a plausible association of ambient air pollutants (AAP) such as nitrogen dioxide (NO_2_), sulfur dioxide (SO_2_), particulate matter (PM) 2.5 and PM_10_ in the viral transmission and related mortality [6–11]. Similarly, meteorological factors such as ambient temperature (AT), relative humidity (RH) etc., have been identified to promote sustained transmission of SARS-CoV-2 in China and Singapore [12, 13].

In India, several studies have highlighted the link between exposure to AAP and its adverse health effects [14, 15]. It is also noteworthy that the air quality index of few urban and non-urban areas of India is high [16–19]. Furthermore, India is the second most affected country by SARS-CoV-2 globally after the USA [20]. Therefore, to formulate control measures and develop policy decisions, it is important to understand the impact of AAP and meteorological factors on SARS-CoV-2 transmission, hospitalisation, severity, and mortality. Against this background, the present study was conducted to examine the association between AAP and meteorological factors that in turn could influence the SARS-CoV-2 transmission and related mortality in India.

## Materials and methods

### Data collection and screening of cities

**Fig. 1:**
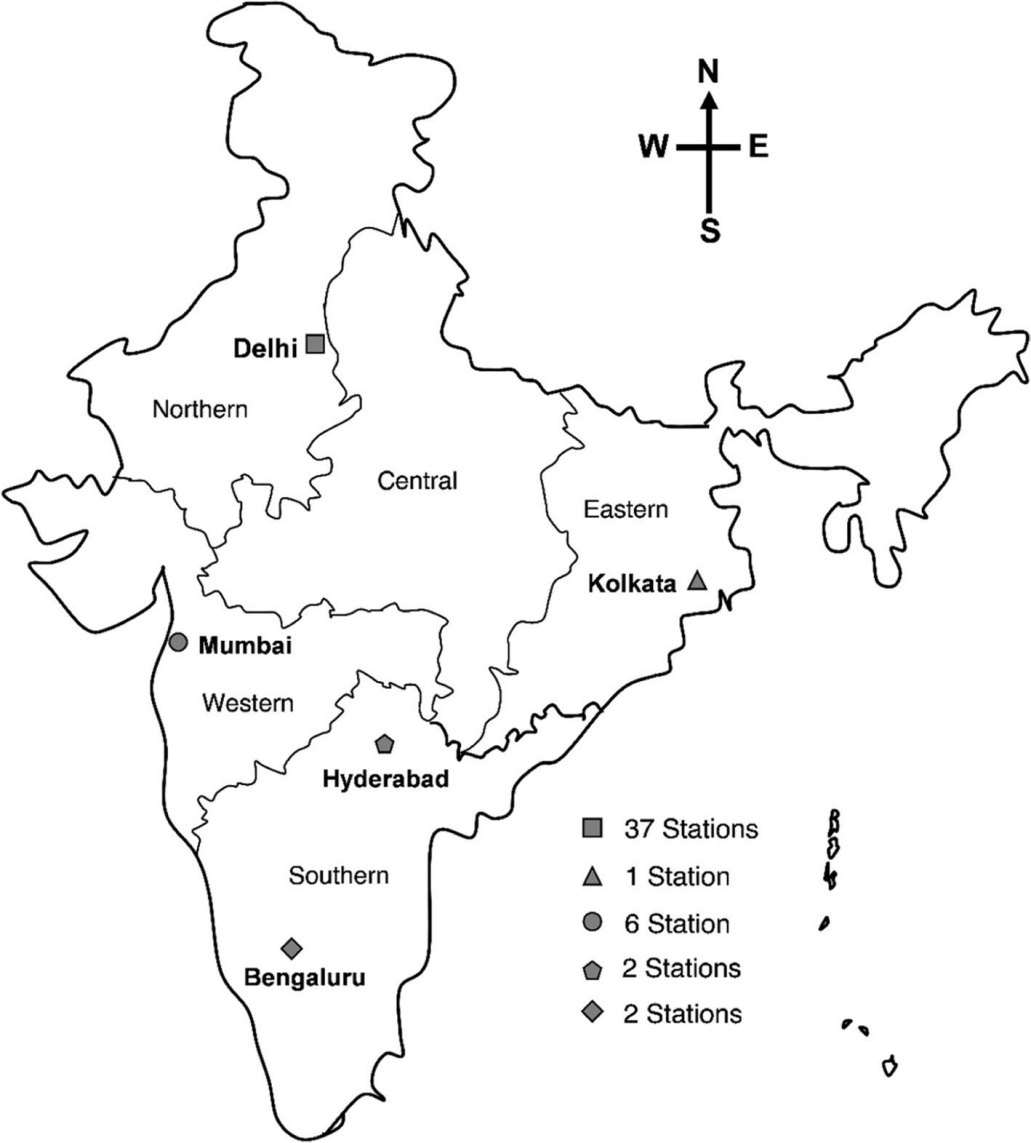
Cities included in the study that located in various zones of India. The number of CPCB monitoring stations used for data collection form each city are given

The study period was divided into three phases, Pre-lockdown (January-March 2020), Phase I (April-June 2020) and Phase II (July- November 2020). The rationale behind such phase separation was anticipated altered air quality across phases owing to the complete lockdown (Phase I) and unlocking (Phase II) enforced in India (Table 1). The present study used daily AAP and meteorological data of India, which are openly accessible from the Indian Central Pollution Control Board’s website (CPCB). In December 2020, 232 active CPCB monitoring stations located in 73 districts were assessed for the period 1^st^ January 2020 to 22^nd^ November 2020. The following four criteria were kept into consideration to ensure the quality of the outcomes; i) stations with availability of at least 80% of the data for the total study duration, ii) cities with AAP level above the acceptable range as per the National Ambient Air Quality Standards (NAAQS), iii) the top ten densely populated cities and iv) cities that come under major zones (East, North, South and West) of the country, as these are the critical factor that might influence the transmission of SARS-CoV-2. Based on such considerations, Delhi, Kolkata, Mumbai, Hyderabad and Bengaluru having 48 stations, were selected for final analysis. The final list of Cities/districts and their stations selected for analyses are depicted in Fig. 1.

**Table 1:** Division of study duration

**Pre-Lock down** (01.01.2020 – 31.03.2020)	**Phase -1** Lockdown to Unlock 1 (01.04.2020- 31.06.2020) **Activities permitted and prohibited**		**Phase -2** Unlock 2- Unlock 6 (01.07.2020 – 22.11.2020) **Activities permitted and prohibited**	
	Lockdown	Total lock down of the country except the movement of frontline workers	Unlock 2 -3	All activities including public and private transportation and industries were permitted except the following outside of the containment zones; Educational Research institutes, International travel, Metro rail and Entertainment places
	Unlock 1	Relaxation for restricted interstate travel, religious places, hospitality services	Unlock 4-6	All kinds of activities were throughout the nation except in the containment zones. Restricted international travel

Indian Council of Medical Research (ICMR) has been archiving COVID-19 testing and diagnosis data in the centralised server, maintained by the Bioinformatics division since March 2020. This online server stores daily individual-level data regarding tests conducted, type of tests, results, socio-economic information, epidemiological and clinical profile of the tested participants etc., at the district level across all states in India. The daily data for tests conducted and positive case counts of Mumbai, Delhi, Kolkata, Hyderabad and Bengaluru were collected using Structured Query Language (SQL) for the outbreak period 1st April 2020 to 22nd November 2020. Further, the CPCB and ICMR data were analysed to understand the correlation between SARS-CoV-2 transmission, and AAP and meteorological factors.

Additionally, an association between long-term AAP exposure (Data collected from CPCB website for the period January 2015 to November 2020) and COVID-19 mortality was explored. The case fatality rate (CFR), indicating the proportion of people who died from COVID-19 among individuals diagnosed with SARS-CoV-2 infection, were calculated to assess mortality. The CFR data was available only for Delhi and Kolkata from the respective State official websites. Hence, they were considered for further analysis. The primary sources of data used in this study are given in Table S1.

#### Screening of Ambient Air Pollutants (AAP) and Meteorological variables

A comprehensive literature review was performed to identify the environmental and meteorological parameters associated with SARS-CoV-2 transmission and mortality. Six pollutants (PM_2.5_, PM_10_, CO, NO_2_, SO_2_, Ozone-O_3_) and four meteorological parameters (Ambient Temperature- AT, Relative Humidity - RH, Rainfall - RF, Wind Speed - WS) were initially identified. However, it was noticed that except for PM_2.5_, PM_10_ and AT, variability in other parameters was minimal in the selected cities. Hence, these three parameters were considered for final analysis. The NAAQS defined by CPCB for AAP are presented in Table S2.

### Data analysis

The extracted data were analysed for kurtosis and asymmetry. As variables were not normally distributed, non-parametric tests were conducted for analysis. The Mann Whitney Wilcoxon test, a non-parametric test for assessing the equality of means in two independent samples, was used to determine and compare the variations in AAP level between Pre-lock down - Phase I and Phase I - Phase II. The Kruskal-Wallis test, a non-parametric test that compares the mean rank of three are more different groups, was used to determine the variations in AAP level of the past six year's data (2015-2020). Further, Kruskal-Wallis Paired Comparisons (Conover) was carried out to know the significance among each paired group. Finally, Spearman and Kendall correlation tests were used to check for association of exposure to AAP and meteorological factors on SARS-CoV-2 transmission and mortality.

#### Adjusted Test Positivity Ratio (ATPR)

Comprehending trends of test positivity ratio (TPR), which is the ratio of the number of positive test results and the number of tests performed, may lead to misinterpretation, as both the numerator and the denominator were changing due to various reasons such as scaling up of testing capacity, changes in testing criteria for COVID-19 and the number of cases detected at the beginning and later over a period of time. Therefore, an adjusted test positivity ratio (ATPR) was estimated to examine the association of AAP and AT factors with SARS-CoV-2 transmission to overcome potential biases due to fluctuation in the aforementioned numerators and denominators.

ATPR on the day 't' was calculated by multiplying reported test positivity with the daily ratio of increase in cases to tests [21] using the formula, ATPR =TPR * Z_t_ where z_t_= r_case_t_/r_test_t_ (where r_case_t_ = C_t_−(C_t_–1) /C_t_–1 is the growth rate of cases and r test_t_ =T_t_−(T_t_–1) /T_t_–1 is the growth rate for tests.)

In order to identify the association of AAP exposure and AT with COVID-19 ATPR, lag values of 7 and 14 days were considered. It is known that the incubation period for COVID-19 is 7.76 days, and hence a lag of 7 days has been considered [22]. Therefore, lag7 was calculated on day 't' by taking PM values for day t-7. Similarly, Lag14 was determined by taking PM values for day t-14. As there might be a lagged association between the exposure and disease outcome, it is essential to take a moving-average approach to determine the lag effect of variables on SARS-CoV-2 transmission [23].

#### Case Fatality Rate (CFR)

In the present study, the effect of long-term exposure to AAP on COVID-19 mortality was analyzed through Case Fatality Rate (CFR). The reported CFR is the proportion of people who died from COVID-19 among individuals diagnosed over a specified period. In this ecological study model, these cities' population was assumed as constant and exposed to AAP for long-term. So, whenever a person who gets infected by COVID-19 had long-term exposure to AAP and also exposed during the course of illness till they die. Thus, to study the six-year cumulative effect of PM_2.5_ and PM_10_ on mortality due to COVID 19, the cumulative average for six years till day 't' was calculated for the period 1^st^ January 2015 to 22^nd^ November 2020.

$$\left[{T}_t,{T}_{t+1}\cdots {T}_n\right]=\left[\frac{\sum_{i=1}^t pm{2.5}_i}{t},\frac{\sum_{i=1}^{t+1} pm{2.5}_i}{t+1}\cdots \frac{\sum_{i=1}^n pm{2.5}_i}{n}\right]$$

Cumulative average = T; Cumulative average on day t = Tt; Cumulative average on day t+1= Tt+1

Cumulative average on day n = Tn; n= 365*5+236=2061. Then, to analyse the correlation between long-term exposure to AAP and COVID 19 CFR Spearman and Kendall correlation test was conducted.

### Ethics approval

The Institutional Ethical Committee (IEC) clearance was obtained from the Central Ethics Committee on Human Research (CECHR), Ref No. NCDIR/BEU/ICMR-CECHR/75/2020.

## Results

The level of air pollutants was analysed in all the five selected cities to understand the variations in AAP levels among different study phases.

The daily average levels of PM_2.5_ and PM_10_ were compared between Pre-lockdown (January-March, 2020) and Phase I (April-June, 2020) and both AAPs were found to be significantly reduced (Mann Whitney Wilcoxon Test, *p*<0.001) during Phase I in all the cities (Table S3, Fig. S1). In Kolkata, Mumbai, Bengaluru and Hyderabad, the daily average of PM_2.5_ got significantly reduced by 351, 293, 203, and 123% in Phase I compared to the Pre-lockdown Phase (Mann Whitney Wilcoxon Test, *p*<0.001; Table S3, Fig. S1). On the other hand, the daily average of PM_10_ levels decreased to 317, 209, 55 and 46%, respectively, in Kolkata, Mumbai, Hyderabad and Bengaluru during Phase I. Interestingly, in Delhi, the ‘Poor’ AAP levels (as per NAAQS of India) observed in the Pre-lock down period (PM_2.5_ 109 μg/m^3^ and PM_10_ 250 μg/m^3^) got drastically reduced to Satisfactory to Moderate level in Phase I (PM_2.5_ 50.71 μg/m^3^ and PM_10_ 119.82 μg/m^3^, respectively). However, the past five-year trend (2015-2019) was different where, the daily average of PM_2.5_ and PM_10_ levels (2015-2019) were higher by 70 and 155% when compared with the same time period in 2020 (Phase I), which was highly significant (Kruskal-Wallis test, *p*<0.0001) (Fig. S2, Table 3).

**Fig. 2:**
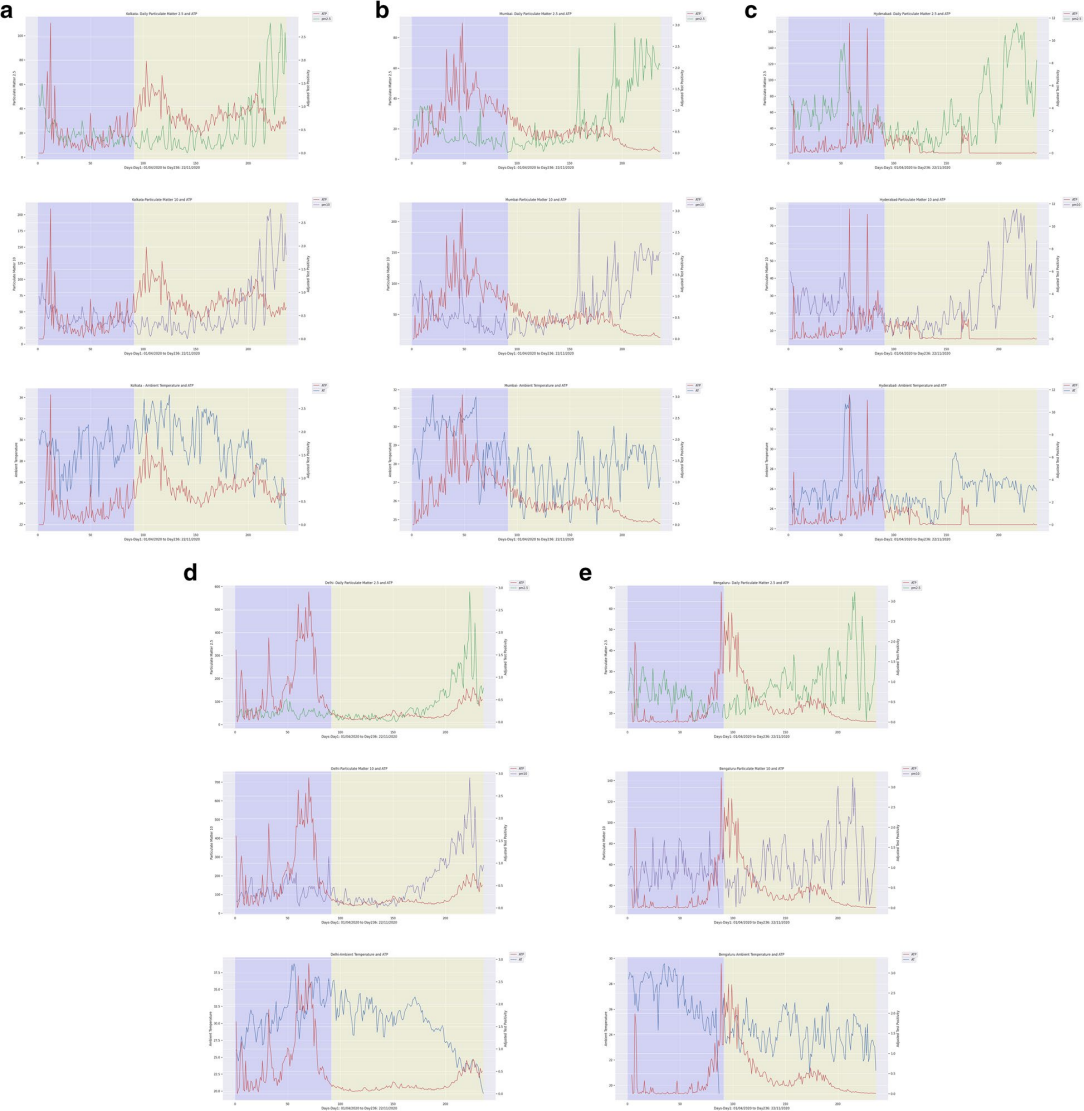
Daily confirmed COVID-19 cases along with Ambient Air Pollutants and Ambient Temperature in Kolkata (**a**), Mumbai (**b**), Hyderabad (**c**), Delhi (**d**) and Bengaluru (**e**) from April 2020 to November 2020

The comparison was made between Phase I (April-June 2020) and Phase II (July-November 2020) to check for the variation in AAP levels. In all selected cities, the daily average of PM_2.5_ and PM_10_ increased in Phase II (Fig. S1). In Delhi, the daily average of PM_2.5_ levels rose (84%) from ‘Satisfactory’ in Phase I (50.71 μg/m^3^) to ‘Poor’ in Phase II (93.81 μg/m^3^). Also, the daily average of PM_10_ levels increased up to 42% in Phase II (Table 2, Fig. 2). The 2015 to 2019 data further suggest this trend of increased AAP levels during July-November (PM_2.5_ 119.6 μg/m^3^, PM_10_ 257.2 μg/m^3^) while compared with April-June (PM_2.5_ 85.4 μg/m^3^, PM_10_ 217.2 μg/m^3^) in this city. However, the AAP level increase during Phase II (2020) were lower compared to Phase I (PM_2.5_ 93.81 μg/m^3^ and PM_10_ 170.55 μg/m^3^) (Table 2, Fig. S1). This was about 26 and 33% of average reduction in PM_2.5,_ and PM_10_ levels, respectively, compared to the past five years and were statistically significant (Kruskal-Walis test, *p*<0.001). Similar to Delhi, considerable increases in the AAP were observed in other cities during Phase II compared to Phase I (Fig. S1, Table 2). About 184, 177, 152 and 105%, respectively, increase in Bengaluru, Mumbai, Hyderabad and Kolkata. The daily average of PM_10_ increased up to 164, 147, 134 and 115% in these cities in Phase II. The Mann Whitney Wilcoxon Test showed that PM_2.5_ had significantly increased (*p* values < 0.05) in the cities of Mumbai and Bengaluru. Likewise, in all cities except for Kolkata, PM_10_ levels were significantly increased in Phase II compared to Phase I. These analyses indicated that pollution levels varied among different Phases of the study. Then, the Ambient Temperature (AT) levels analysed in all five cities for Phase I and Phase II (Table S4). The daily average of *mean* AT was seen as lowest in Bengaluru (24.03°C) and highest in Delhi (31.53°C). Similarly, the highest and lowest daily average of *maximum* AT was seen in Bengaluru (24.04°C) and Delhi (35.08°C), respectively.

**Table 2:** Descriptive statistical analysis of Particulate Matters (PM) and Ambient Temperature (AT) data

City	Study period	Variables	Minimum	Maximum	Mean	Standard Deviation
Bengaluru	Phase 1	PM10	19	92	52.99	13.904
		PM2.5	6	32	18.06	6.431
		AT	19	30	26.9	1.902
	Phase 2	PM10	19	143	60.63	24.308
		PM2.5	7	68	23.11	11.751
		AT	21	27	24.05	1.24
	Total	PM10	19	143	57.76	21.299
		PM2.5	6	68	21.21	10.363
		AT	19	30	25.12	2.053
Delhi	Phase 1	PM10	38	302	119.82	45.7
		PM2.5	21	112	50.71	18.055
		AT	24	39	31.53	3.35
	Phase 2	PM10	29	723	170.55	141.277
		PM2.5	11	577	93.81	99.884
		AT	20	36	29.7	3.687
	Total	PM10	29	723	151.2	117.115
		PM2.5	11	577	77.37	81.973
		AT	20	39	30.4	3.664
Hyderabad	Phase 1	PM10	22	146	61.69	8.039
		PM2.5	8	44	24.39	24.977
		AT	23	35	26.37	2.644
	Phase 2	PM10	9	171	61.22	21.093
		PM2.5	5	80	28.2	44.275
		AT	22	30	25.66	1.433
	Total	PM10	9	171	61.4	17.391
		PM2.5	5	80	26.75	38.024
		AT	22	35	25.93	2.009
Kolkata	Phase 1	PM10	15	95	39.57	14.101
		PM2.5	7	60	19.3	9.801
		AT	24	32	29.02	1.78
	Phase 2	PM10	8	209	46.67	41.026
		PM2.5	3	111	21.66	21.119
		AT	25	34	30.18	2.131
	Total	PM10	8	209	43.81	33.056
		PM2.5	3	111	20.7	17.466
		AT	24	34	29.7	2.07
Mumbai	Phase 1	PM10	17	108	47.37	21.296
		PM2.5	6	35	17.03	7.659
		AT	26	32	29.47	1.235
	Phase 2	PM10	11	221	71.04	45.965
		PM2.5	4	90	28.76	22.409
		AT	25	30	27.53	1.216
	Total	PM10	11	221	62.01	40.101
		PM2.5	4	90	24.28	19.096
		AT	25	32	28.27	1.543

The association of AAP exposure with SARS-CoV-2 transmission was analysed by performing Spearman and Kendall rank correlation for lag 0, lag 7 and lag 14 days in Phase I and Phase II (Fig. 2, Table 3). This non-parametric analysis showed a positive correlation for the daily average of *mean* PM_2.5_ and PM_10_ with COVID-19 ATPR (*p*<0.001 for lag 0, lag 7, and lag 14) during Phase II and no correlation during Phase I in Delhi. The AAP level was not positively associated with the SARS-CoV-2 transmission in both Phase I and Phase II in other cities. In Bengaluru, Hyderabad and Mumbai, a negative correlation was observed for PM_2.5_ and PM_10_ with COVID-19 ATPR (*p*<0.001 for lag 0, lag 7, and lag 14). Interestingly, no correlation was observed for Kolkata for the total study period. When the analysis was conducted with a daily average of *maximum* PM2.5 and PM10, the correlation coefficient was similar to the daily average of mean PMs (Table S5).

**Table 3:** Correlation coefficient analysis between COVID-19 ATPR, and daily average of mean Particulate Matters (PM) and Ambient Temperature (AT). (‘+’ values are considered as positively significant and ‘-‘ values are positively significant, *p<0.05, **p<0.01)

Study Period	Variables	Lag	Bengaluru		Delhi		Hyderabad		Kolkata		Mumbai	
			K	S	K	S	K	S	K	S	K	S
**Phase I**	AT	No lag	-.373**	-.531**	.240**	.348**	.267**	.375**	-0.079	-0.123	0.165*	0.255*
		Lag 7	-.343**	-.494**	.338**	.486**	0.031	0.033	-0.034	-0.045	-0.019	-0.0149
		Lag 14	-.301**	-.440**	.263**	.385**	0.146*	0.213*	0.001	-0.008	-0.112	-0.1614
	PM 2.5	No lag	-.308**	-.465**	0.057	0.081	-.198^**^	-.293**	-0.0369	-0.032	-.337^**^	-.476**
		Lag 7	-.240**	-.343**	-0.055	-0.089	-.356^**^	-.515**	0.01979	0.045	-.259^**^	-.368**
		Lag 14	-.185*	-.273*	0.049	0.086	-.345^**^	-.509**	-0.0128	-0.028	-.209^**^	-.289**
	PM 10	No lag	-0.0262	-0.0582	0.073	0.104	-0.120	-0.1715	0.03128	0.045	-.237^**^	-.343**
		Lag 7	-0.0544	-0.0777	0.012	0.014	-.294^**^	-.448**	0.04731	0.073	-.178^*^	-.259*
		Lag 14	-.222**	-.321**	0.002	0.007	-.317^**^	-.481**	-0.1329	-0.197	-.249^**^	-.362**
**Phase II**	AT	No lag	0.086	0.121	-.370^**^	-.536^**^	-.257^**^	-.420^**^	0.117	0.165	-.169^**^	-.246^**^
		Lag 7	0.083	0.117	-.278^**^	-.405^**^	-.255^**^	-.413^**^	0.068	0.102	-.160^**^	-.229^**^
		Lag 14	0.098	0.142	-.222^**^	-.323^**^	-.259^**^	-.415^**^	0.065	0.108	-0.091	-0.149
	PM 2.5	No lag	-.370^**^	-.489^**^	.367^**^	.528^**^	-.407^**^	-.591^**^	-0.058	-0.091	-.426^**^	-.607^**^
		Lag 7	-.298^**^	-.399^**^	.351^**^	.529^**^	-.492^**^	-.694^**^	0.005	0.015	-.392^**^	-.558^**^
		Lag 14	-.236^**^	-.324^**^	.326^**^	.492^**^	-.409^**^	-.605^**^	0.103	0.148	-.367^**^	-.533^**^
	PM 10	No lag	-.250^**^	-.332^**^	.372^**^	.537^**^	-.398^**^	-.586^**^	-0.095	-0.148	-.409^**^	-.577^**^
		Lag 7	-.155^**^	-.208^*^	.342^**^	.522^**^	-.476^**^	-.682^**^	-0.038	-0.049	-.398^**^	-.561^**^
		Lag 14	-0.058	-0.075	.296^**^	.450^**^	-.378^**^	-.566^**^	0.073	0.096	-.378^**^	-.544^**^
**Total period**	AT	No lag	-.256^**^	-.384^**^	-0.021	-0.019	-0.069	-0.119	0.103	0.156	.213^**^	.338^**^
		Lag 7	-.273^**^	-.407^**^	.088^*^	.146^*^	-.146^**^	-.227^**^	0.071	0.107	.133^**^	.205^**^
		Lag 14	-.247^**^	-.379^**^	.164^**^	.256^**^	-.209^**^	-.332^**^	0.030	0.044	0.081	0.121
	PM 2.5	No lag	-.217^**^	-.305^**^	.190^**^	.295^**^	-.279^**^	-.406^**^	-0.031	-0.034	-.376^**^	-.538^**^
		Lag 7	-.100^*^	-.136^*^	.129^**^	.205^**^	-.380^**^	-.544^**^	0.045	0.076	-.386^**^	-.549^**^
		Lag 14	-0.017	-0.015	.099^*^	.169^*^	-.352^**^	-.518^**^	0.092	0.137	-.384^**^	-.553^**^
	PM 10	No lag	-.100^*^	-.139^*^	.218^**^	.332^**^	-.230^**^	-.339^**^	-0.024	-0.031	-.343^**^	-.496^**^
		Lag 7	-0.053	-0.076	.157^**^	.251^**^	-.326^**^	-.476^**^	0.035	0.055	-.360^**^	-.519^**^
		Lag 14	-0.037	-0.059	.104^*^	.175^**^	-.291^**^	-.437^**^	0.064	0.096	-.380^**^	-.548^**^

Further, the association between SARS-CoV-2 transmission and AT were analysed. The data showed both significant positive and negative correlations between AT (daily average of the maximum and mean AT) and COVID-19 ATPR among cities during both Phases (Table 3). A negative correlation between COVID-19 ATPR and AT (*p*<0.001 for lag 0, lag 7, and lag 14) was seen in Bengaluru during Phase I and in the rest of the cities during Phase II except Kolkata. For Delhi, Hyderabad and Mumbai, a positive correlation between COVID-19 ATPR and AT (*p*<0.01 for lag 0, lag 7 and lag 14) were observed (Fig. 2). Notably, the correlation coefficient for both the daily average of *maximum* AT and *mean* AT were similar (Table S5).

**Fig. 3:**
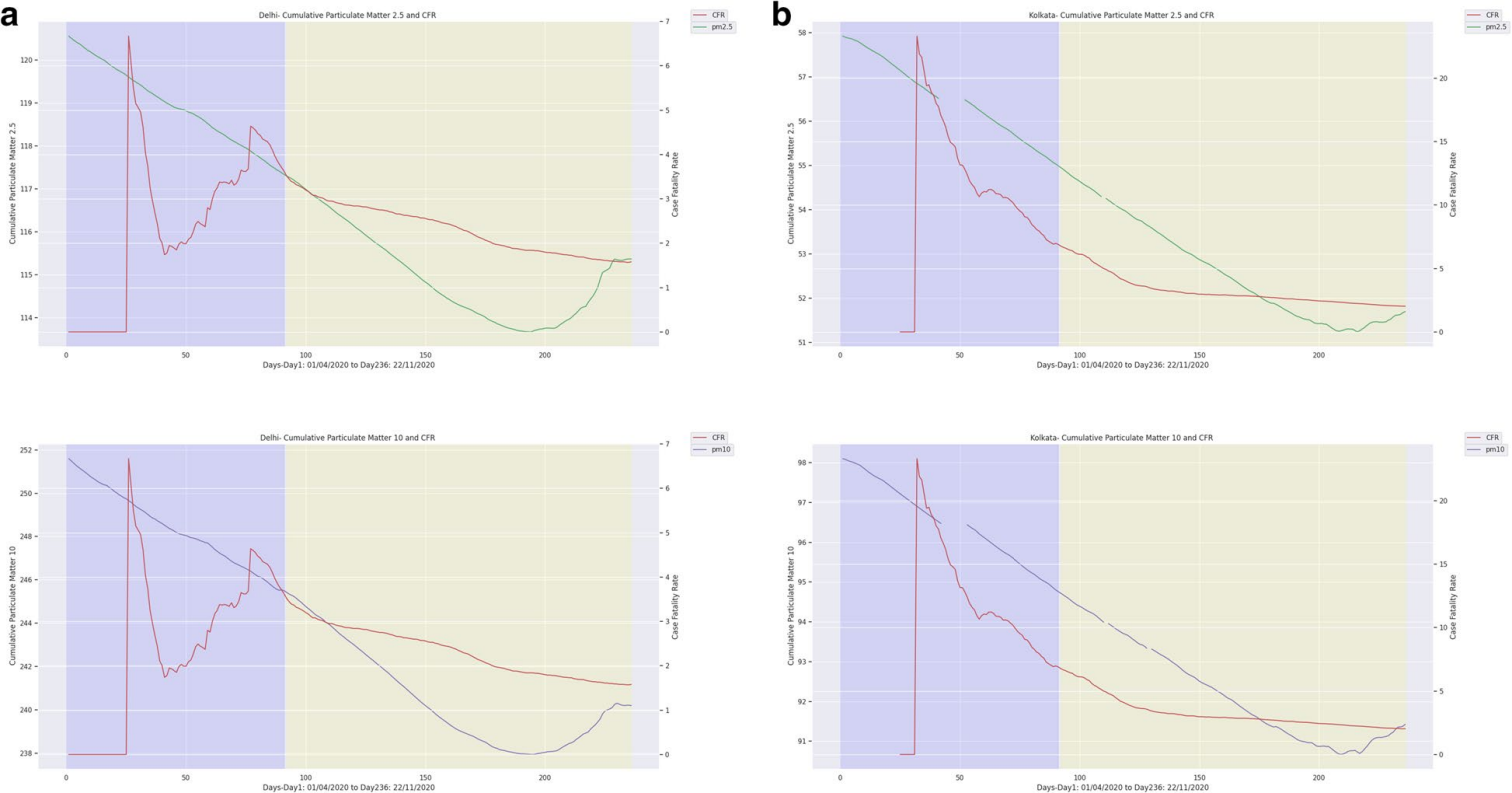
Daily case fatality rate (CFR) of COVID-19 and along with Ambient Air Pollutants in Delhi **(a)** and Kolkata **(b)** from April 2020 to November 2020

To investigate the effect of long-term exposure to PM_2.5_ and PM_10_ on COVID-19 related mortality, the correlation coefficient between COVID-19 CFR and AAP were computed. In Delhi, the last six year's (January 2015 to December 2020) daily average levels of PM_2.5_ (102 μg/m^3^) and PM_10_ (237 μg/m^3^) were found to be exceeding the permissible limit according to NAAQS in India. In Kolkata, it was 29 and 58μg/m^3^, respectively, for PM_2.5_ and PM_10_. The Spearman and Kendall rank analysis showed that the correlation coefficients for PM_2.5_ were 0.64 (99% CI, *p*<0.01) and 0.77 (99% CI, *p*<0.01), respectively for Delhi and Kolkata, indicating significant positive correlation between AAP and COVID-19 CFR (Table 4). Similarly, for PM_10,_ the correlation coefficients were 0.78 (99% CI, *p*<0.01) and 0.80 (99% CI, *p*<0.01) for Delhi and Kolkata, respectively (Fig. 3). These results indicated possible association between long term exposure to AAP and COVID-19 related deaths.

**Table 4.** Correlation coefficient analysis between COVID19-CFR and Particulate Matters (PM). (‘+’ values are considered as positively significant and ‘-‘ values are positively significant, *p<0.05, *p<0.01)

Period	Cities	PM_**2.5**_	PM_**10**_	PM_**2.5**_	PM_**10**_
		Spearman_rho		Kendall’s_tau	
Phase 1	Delhi	-0.25**	-0.25**	-0.27*	-0.27**
	Kolkata	0.33*	0.33*	0.51**	0.51**
Phase 2	Delhi	0.71**	0.76*	0.60**	0.63**
	Kolkata	1.00**	0.95**	0.99**	0.86**
Total study period	Delhi	0.64**	0.70**	0.77**	0.78**
	Kolkata	0.78**	0.77**	0.80**	0.78**

## Discussion

The present study found that PM_2.5_ and PM_10_ levels were significantly reduced in all the selected cities during Phase I (Fig. S1, Table S1). The past six years’ data (2015-2020) of Delhi also indicated that lower PM_2.5_ and PM_10_ levels were recorded during April-June 2020 (Fig. S2). The shutdown of anthropogenic activities like industries, transportation, infrastructure construction activities etc., might be the cause for this reduced emission of Particulate Matters and therefore improved air quality [24]. Similar findings were also noticed by other investigations conducted in Delhi, Mumbai, Chennai, Kolkata, and Bengaluru [25–28]. In addition to India, China, France, Italy, Spain, and Germany also enforced restrictions that lead to a drastic reduction in PM_2.5_ and PM_10_ during lockdown [29–32].

Unlock phases were initiated from June 2020 in the country; however, pollutants continued to drop till August 2020 due to restricted transportation and other industrial activities (Fig. S1, Fig. S1, Table S1). In all five cities, the PM_2.5_ level was <40 μg/m^3^ as per the NAAQS of India during July and August 2020. In the same period, the PM_2.5_ level fell below the NAAQS of India, i.e. 34.56 and 26.58μg/m^3^ for the first time in the last six years in Delhi. In contrast, PM_2.5_ and PM_10_ levels significantly increased in the later period of Phase II as a result of increased anthropogenic activities in the country. AAP levels reached a high level in November 2020, where more than 150% increase was observed in all cities, especially a 300% hike in Delhi compared to Phase I (Fig. 2). Altogether, these findings evidenced that the lockdown measures imposed in most countries to contain the spread of COVID-19 infection reduced the air pollutants that resulted in improved air quality. However, PM_2.5_ and PM_10_ levels increased upon the ease of lockdown, as shown in our study.

In order to explore the relationship between AAP and SARS-CoV-2 transmission, the correlation between Particulate Matters level and COVID-19 ATPR was analysed for Phase I and Phase II (Table 3, Fig. 2). Interestingly, we observed that when the average daily PMs were ‘moderate to poor’ as per the NAAQS category (PM_2.5_ 61-120 μg/m^3^; PM_10_ 101-350 μg/m^3^), there was a positive association between AAP and SARS-CoV-2 transmission. Evidently, in Delhi during Phase II, the daily average of PM_2.5_ (93.81 μg/m^3^) and PM_10_ (170.55 μg/m^3^) were in the ‘poor’ and ‘moderate’ range respectively, thus, a strong positive correlation was identified (Table 3). Similarly, studies conducted across the globe noticed a strong association between AAP and COVID-19 cases, especially with the increased PMs level [29, 33–38]. However, in Mumbai, Kolkata, Hyderabad and Bengaluru, the PM_2.5_ and PM_10_ were in the ‘Good’ to ‘Satisfactory’ category (PM_2.5_ 0-60 μg/m^3^; PM_10_ 0-100 μg/m^3^) and, a negative or no correlation was observed. Notably, a study conducted in Maharashtra (India) obtained similar result [28]. Overall, observations from our study and others show that increased Particulate Matters beyond the moderate level are positively associated with SARS-CoV-2 transmission. Yet, further intensive experimental studies are required to confirm the mechanism involved in such transmission.

Further, the association between the AT (daily average of mean AT and maximum AT) and COVID-19 ATPR were analysed (Table 3, Fig. 2). The correlation analysis showed both positive and negative associations between the daily average of mean AT and COVID-19 ATPR. Delhi, Hyderabad, and Mumbai showed a positive correlation for AT in Phase I and a negative correlation in Phase II. Similar studies conducted in Mumbai and Delhi agree with our results [28, 39]. On the other hand, Bengaluru showed a negative correlation in Phase I and no correlation in Phase II. In Kolkata, no correlation was observed in both Phases. Similar studies conducted in other countries showed positive, negative and heterogeneous associations between temperature and SARS-CoV-2 transmission [28, 40, 41]. In addition, the correlation analysis between the daily average of maximum AT and COVID-19 ATPR showed similar results in both phases because of the similar pattern of variation between mean and maximum AT in all the five cities. The varying trend results observed for AT might be influenced by confounders that could interplay with COVID-19 transmission dynamics. Overall, the present investigation did not indicate any association between temperature and SARS-CoV-2 transmission.

The effect of long-term exposure to AAP on COVID-19 related mortality was studied (Fig. S1). When analysed for the correlation coefficient (r), this long-term exposure to PMs showed a strong positive association with the COVID-19 CFR in Delhi (PM_2.5_ r-0.64, PM_10_ r-0.77) and Kolkata (PM_2.5_ r-0.78, PM_10_ r-0.80) for the entire study duration (Fig. 3, Table 4). Studies conducted in 22 cities of India and other countries, namely Italy, the USA, China, England and France also observed similar associations [42–47]. Importantly, researchers are further investigating to elucidate the threshold level of PMs beyond which they could be associated with COVID-19 mortality. A multicentric study conducted in France proposed such threshold levels of PM_2.5_ (15 μg/m^3^ ± 2) and PM_10_ (25 μg/m^3^ ± 4) [44]. Notably, in Delhi and Kolkata, the PM_2.5_ and PM_10_ levels were significantly high compared to these ranges (Table 4, Fig. 3). Therefore, the present study indicates that long-term exposure to PMs is associated with COVID-19 related mortality, possibly enhancing the host susceptibility to the SARS-CoV-2 infection. However, to prove the biological plausibility of this association, strong epidemiological and experimental studies are needed [48].

Despite generating findings of public health importance, our study has certain limitations as follows. Firstly, the data used in this study were not primarily collected for the interrogation. Instead, we used secondary data obtained from the Indian Meteorological Department. Secondly, the daily average data used for analyses may mask more complicated relationships between the disease as outcome, maximum ambient temperature, duration of the temperature, and exposure to high pollution. Thirdly, it is not possible to link exposure with the disease in individuals as those may not be the same in the exposed population. Hence, caution is needed when applying grouped results to individual level. Fourthly, as COVID- 19 is contagious and primarily affected by various confounding factors including personal hygiene, host genotype, population mobility, health infrastructure, environmental determinants, and people’s adherence to covid appropriate behaviour, a comprehensive investigation is essential to understand the association explicitly. As our study could not adjust for these factors due to the paucity of relevant data, within these confines, our findings should be taken as hypothesis generating rather than as confirmatory.

## Conclusion

The present study found that Particulate Matters' level considerably declined during the lockdown period in all the five selected cities. However, they started increasing at the later period of the Unlocking Phase. Interestingly, whenever the level of Particulate Matters exceeded the permissible range, there was a positive association between air pollutants and SARS-CoV-2 transmission, as evidenced in Delhi. Interestingly, in cities such as Mumbai, Kolkata, Bengaluru and Hyderabad, where satisfactory levels of particulate matter were recorded, since association lacking. In addition, the long term exposure to particulate matters showed a positive correlation with COVID-19 related mortality, which was demonstrated with the past six-year data of Delhi and Kolkata. Together, our study provides preliminary evidence that moderate to highly polluted cities are more likely to be associated with the transmission of SARS-CoV-2 infection related lethal outcome. Thus, future studies must be conducted to determine their threshold level to minimise their transmission. Overall, this study suggested that the level of ambient air pollutants have impact on SARS-CoV-2 morbidity and mortality [37, 43].

## Supplementary Information

**Additional file 1: Figure S1.** Monthly average Ambient Air Pollutants levels among pre-lockdown (January to March 2020), Phase I (April to June 2020) and Phase II (July to November 2020) in Kolkata (**a**), Mumbai (**b**), Hyderabad (**C**), Delhi (**d**) and Bengaluru (e). **Figure S2.** Six-year trend of ambient air pollutants in Delhi (**a**) and Kolkata (**b**). **Table S1.** List of official sources used for data collection in this study. **Table S2.** Categories of Ambient Air Pollutants (AAP) as per their safety levels determined by NAAQS, India. **Table S3.** Mann Whitney Wilcoxon Test results of Particulate Matters (PM) levels among group variables (Pre-lockdown and Phase I, Phase I and Phase II) (*p<0.05, **p<0.01, *** *p*<0.001, # *p*<0.0001). **Table S4.** Daily average of maximum Ambient Temperature (AT) and mean AT. **Table S5.** Correlation coefficient analysis between COVID-19 ATPR, daily average of mean Particulate Matters (PM) and Ambient Temperature (AT). (‘+’ values are considered as positively significant and ‘-‘ values are positively significant, *p<0.05, **p<0.01).

## Data Availability

All data are publicly available, with sources described in the manuscript except for COVID-19 data as it is the restricted access datasets.

## Abbreviations

COVID-19: Coronavirus disease 2019
SARS-CoV-2: Severe Acute Respiratory Syndrome Coronavirus 2
SARS: Severe Acute Respiratory Syndrome
MERS: Middle East Respiratory Syndrome
COPD: Chronic obstructive pulmonary disease
AAP: Ambient air pollutants
NO_2_: Nitrogen dioxide
SO_2_: Sulfur dioxide
PM: Particulate matter
CPCB: Central Pollution Control Board
CFR: Case fatality rate
Ozone: O_3_
CO: Carbon monoxide
AT: Ambient Temperature
RH: Relative Humidity
RF: Rainfall
WS: Wind Speed
ICMR: Indian Council of Medical Research
WHO: World Health Organization
ATPR: Adjusted Test Positivity Ratio
CECHR: Central Ethics Committee on Human Research
NAAQS: National Ambient Air Quality Standards
ACE-2: Angiotensin-converting enzyme 2
