# Supplementary material for: Association between ambient air pollutants and meteorological factors with SARS-CoV-2 transmission and mortality in India: an exploratory study
Source: Environ Health. 2021 Nov 19;20:120. doi: 10.1186/s12940-021-00804-0 (PMC8601781; doi:10.1186/s12940-021-00804-0)
Supplement: Supplementary file 1 — Additional file 1: Figure S1. Monthly average Ambient Air Pollutants levels among pre-lockdown (January to March 2020), Phase I (April to June 2020) and Phase II (July to November 2020) in Kolkata (a), Mumbai (b), Hyderabad (C), Delhi (d) and Bengaluru (e). Figure S2. Six-year trend of ambient air pollutants in Delhi (a) and Kolkata (b). Table S1. List of official sources used for data collection in this study. Table S2. Categories of Ambient Air Pollutants (AAP) as per their safety levels determined by NAAQS, India. Table S3. Mann Whitney Wilcoxon Test results of Particulate Matters (PM) levels among group variables (Pre-lockdown and Phase I, Phase I and Phase II) (*p<0.05, **p<0.01, *** p<0.001, # p<0.0001). Table S4. Daily average of maximum Ambient Temperature (AT) and mean AT. Table S5. Correlation coefficient analysis between COVID-19 ATPR, daily average of mean Particulate Matters (PM) and Ambient Temperature (AT). (‘+’ values are considered as positively significant and ‘-‘ values are positively significant, *p<0.05, **p<0.01). [file 12940_2021_804_MOESM1_ESM.docx]

**Supplementary Material**

**Title:**

Association between Ambient Air Pollutants and Meteorological factors with SARS-CoV-2 transmission and mortality in India: An exploratory study.

**Authors:**

Sumit Aggarwal^1#^, Sivaraman Balaji^1#^, Tanvi Singh^1^, Geetha R. Menon^2^, Sandip Mandal^1^, Madhumathi J^1^, Nupur Mahajan^1^, Jasmine Kaur^1^, Harpreet Singh^1^, Kiran Rade^3^, Samiran Panda^1*^.

**Author’s Affiliations:**

1. Division of Epidemiology and Communicable Diseases, Indian Council of Medical Research-Headquarters, New Delhi – 110029, India
2. Indian Council of Medical Research-National Institute of Medical Statistics, New Delhi – 110029, India.
3. World Health Organization, New Delhi- 110002 India.

**# Authors equally contributed**

*** Corresponding Author**

Dr. Samiran Panda, Scientist G and Head, Division of Epidemiology and Communicable Diseases, Indian Council of Medical Research-Headquarters, New Delhi – 110029, India. Email: [pandasamiran@gmail.com](mailto:pandasamiran@gmail.com), Phone No: +91-11-26588296.

**Supplementary Figures**

**Figure S1.** Monthly average Ambient Air Pollutants levels among pre-lockdown (January to March 2020), Phase I (April to June 2020) and Phase II (July to November 2020) in Kolkata **(a)**, Mumbai **(b)**, Hyderabad **(C)**, Delhi **(d)** and Bengaluru **(e)**.

**b**

**a**

**d**

**c**

**e**

**Figure S2.** Six-year trend of ambient air pollutants in Delhi **(a)** and Kolkata **(b).**

**a**


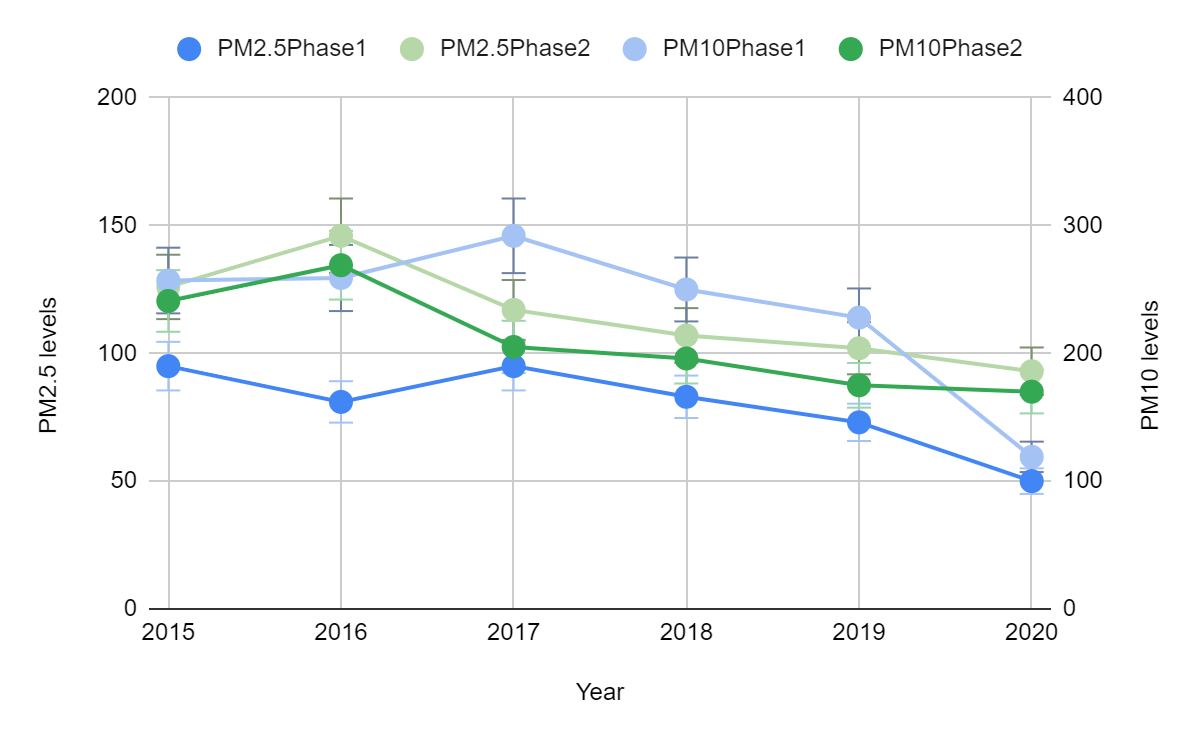

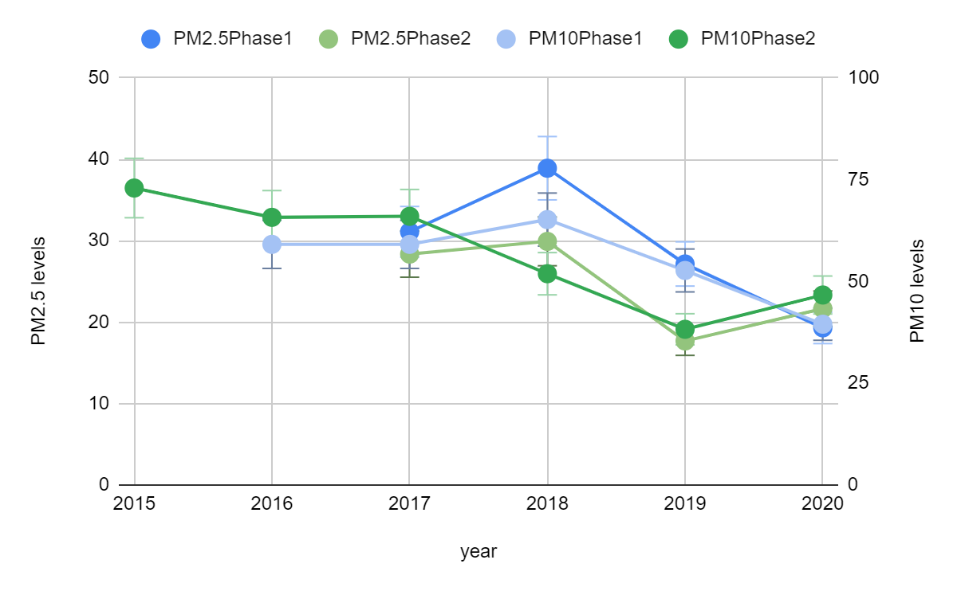


**b**

**Supplementary Tables**

**Table S1.** List of official sources used for data collection in this study.

| **Data particulars** | **Sources** |
| --- | --- |
| Environmental data for PM 2.5, PM 10 and CO emissions | https://cpcb.nic.in/ |
| Meteorological data - Temperature and Relative humidity | <https://cpcb.nic.in/> |
| Population data - population density and population | <https://censusindia.gov.in/>, <http://data.un.org/Search.aspx?q=india> |
| COVID data - for test positivity | ICMR - ISRM database |
| Mortality data –Delhi | http://health.delhigovt.nic.in/wps/wcm/connect/  doit_health/Health/Home/Covid19/Bulletin+Apr+2020 |
| Mortality data- Kolkata | http://health.delhigovt.nic.in/wps/wcm/connect/  doit_health/Health/Home/Covid19/Bulletin+Apr+2020 |

**Table S2.** Categories of Ambient Air Pollutants (AAP) as per their safety levels determined by NAAQS, India.

| **Category** | **PM 2.5** | **PM 10** | **NO2** | **SO2** | **CO** | **Ozone** | **Score** |
| --- | --- | --- | --- | --- | --- | --- | --- |
| **Good** | 0-30 | 0-50 | 0-40 | 0-40 | 0-1.0 | 0-50 | 1 |
| **Satisfactory** | 31-60 | 51-100 | 41-80 | 41-80 | 1.1-2.0 | 51-100 | 2 |
| **Moderate** | 61-90 | 101-250 | 81-180 | 81-380 | 2.1-10 | 101-168 | 3 |
| **Poor** | 91-120 | 251-350 | 181-280 | 381-800 | 10-17 | 169-208 | 4 |
| **Very poor** | 121-250 | 351-430 | 281-400 | 801-1600 | 17-34 | 209-748 | 5 |
| **Severe** | >250 | >430 | >400 | >1600 | >34 | >748 | 6 |

**Table S3.** Mann Whitney Wilcoxon Test results of Particulate Matters (PM) levels among group variables (Pre-lockdown and Phase I, Phase I and Phase II) (**p*<0.05, ***p*<0.01, *** *p*<0.001, # *p*<0.0001).

| **Variables** | **Cities** | **Asymp.sig** |
| --- | --- | --- |
| **Pre-lockdown and Phase I** | | |
| **PM_2.5_** | Delhi | 0.0001 |
|  | Bengaluru | 0.0001 |
|  | Mumbai | 0.001 |
|  | Hyderabad | 0.0001 |
|  | Kolkata | 0.0001 |
| **PM_10_** | Delhi | 0.0001 |
|  | Bengaluru | 0.0001 |
|  | Mumbai | 0.0001 |
|  | Hyderabad | 0.0001 |
|  | Kolkata | 0.0001 |
| **Phase I and Phase II** | | |
| **PM_2.5_** | Delhi | 0.239 |
|  | Bengaluru | 0.001 |
|  | Mumbai | 0.006 |
|  | Hyderabad | 0.089 |
|  | Kolkata | 0.351 |
| **PM_10_** | Delhi | 0.003 |
|  | Bengaluru | 0.047 |
|  | Mumbai | 0.0001 |
|  | Hyderabad | 0.007 |
|  | Kolkata | 0.631 |

**Table S4.** Daily average of maximum Ambient Temperature (AT) and mean AT.

| **Duration** | **AT range** | **Delhi** | **Bengaluru** | **Mumbai** | **Hyderabad** | **Kolkata** |
| --- | --- | --- | --- | --- | --- | --- |
| **Phase I** | **Mean** | 31.53 | 26.30 | 29.47 | 26.37 | 28.70 |
|  | **Maximum** | 35.08 | 26.30 | 30.40 | 29.40 | 28.97 |
| **Phase II** | **Mean** | 29.70 | 24.05 | 27.53 | 25.66 | 28.60 |
|  | **Maximum** | 35.04 | 24.03 | 28.47 | 29.11 | 28.65 |
| **Total** | **Mean** | 30.40 | 25.12 | 28.27 | 25.93 | 28.63 |
|  | **Maximum** | 35.09 | 24.91 | 29.22 | 29.22 | 28.77 |

**Table S5.** Correlation coefficient analysis between COVID-19 ATPR, daily average of mean Particulate Matters (PM) and Ambient Temperature (AT). (‘+’ values are considered as positively significant and ‘-‘ values are positively significant, **p*<0.05, ***p*<0.01).

| **Study Period** | **Variables** | **Lag** | **Bengaluru** | | **Delhi** | | **Hyderabad** | | **Kolkata** | | **Mumbai** | |
| --- | --- | --- | --- | --- | --- | --- | --- | --- | --- | --- | --- | --- |
|  |  |  | K | S | K | S | K | S | K | S | K | S |
| **Phase I** | AT | No lag | -.399** | -.560** | .262** | .389** | .422** | .613** | -0.092 | -0.139 | 0.144* | 0.233* |
|  |  | Lag 7 | -.410** | -.583** | .352** | .502** | 0.213* | 0.341* | -0.017 | -0.024 | -0.009 | -0.013 |
|  |  | Lag 14 | -.325** | -.477** | .260** | .373** | 0.158* | 0.227* | -0.0024 | -0.047 | -0.101 | -0.147 |
|  | PM 2.5 | No lag | -.337** | -.499** | 0.065 | 0.105 | -.117 | -.171 | -0.032 | -0.029 | -.299^**^ | -.403** |
|  |  | Lag 7 | -.347** | -.475** | -0.011 | -0.021 | -.277^**^ | -.404** | 0.023 | 0.049 | -.161^**^ | -.236** |
|  |  | Lag 14 | -.194** | -.279** | 0.126 | 0.198 | -.265^**^ | -.389** | -0.025 | -0.041 | -.0.189^**^ | -.215** |
|  | PM 10 | No lag | -0.068 | -0.119 | 0.064 | 0.090 | -0.014 | -0.020 | 0.014 | 0.021 | -.295^**^ | -.378** |
|  |  | Lag 7 | -0.168* | -0.251* | -0.017 | -0.022 | -.195^**^ | -.295** | -0.015 | -0.028 | -.186^*^ | -.287* |
|  |  | Lag 14 | -.200** | -.301** | 0.053 | 0.075 | -.230^**^ | -.355** | -0.175 | -0.259 | -.251*^*^ | -.343** |
| **Phase II** | AT | No lag | 0.086 | 0.121 | -.230^**^ | -.273^**^ | -.133^**^ | -.202^**^ | .151 | 0.213 | -.219^**^ | -.326^**^ |
|  |  | Lag 7 | 0.087 | 0.118 | -.0.089 | -0.128 | -.143^**^ | -.209^**^ | 0.050 | 0.079 | -.216^**^ | -.320^**^ |
|  |  | Lag 14 | 0.067 | 0.086 | -.0.026 | -0.036 | -.178^**^ | -.257^**^ | 0.007 | 0.030 | -0.119* | -0.201* |
|  | PM 2.5 | No lag | -.370^**^ | -.489^**^ | .340^**^ | .498^**^ | -.384^**^ | -.556^**^ | -0.074 | -0.114 | -.422^**^ | -.589^**^ |
|  |  | Lag 7 | -.298^**^ | -.399^**^ | .360^**^ | .470^**^ | -.474^**^ | -.677^**^ | 0.014 | 0.012 | -.404^**^ | -.580^**^ |
|  |  | Lag 14 | -.248^**^ | -.336^**^ | .283^**^ | .436^**^ | -.389^**^ | -.528^**^ | 0.095 | 0.130 | -.396^**^ | -.570^**^ |
|  | PM 10 | No lag | -.250^**^ | -.332^**^ | .396^**^ | .569^**^ | -.380^**^ | -.557^**^ | -0.109 | -0.170 | -.360^**^ | -.503^**^ |
|  |  | Lag 7 | -.151^**^ | -.201^*^ | .351^**^ | .593^**^ | -.458^**^ | -.662^**^ | -0.048 | -0.070 | -.364^**^ | -.510^**^ |
|  |  | Lag 14 | -0.074 | -0.101 | .292^**^ | .439^**^ | -.356^**^ | -.538^**^ | 0.059 | 0.063 | -.368^**^ | -.517^**^ |
| **Total Period** | AT | No lag | -.267^**^ | -.398^**^ | -0.011 | -0.038 | -0.194* | -.300* | 0.091 | 0.139 | .162^**^ | .256^**^ |
|  |  | Lag 7 | -.290^**^ | -.427^**^ | .124^*^ | .194^*^ | -.185^**^ | -.288^**^ | 0.045 | 0.070 | .090^**^ | .136^**^ |
|  |  | Lag 14 | -.247^**^ | -.379^**^ | .193^**^ | .294^**^ | -.182^**^ | -.273^**^ | -0.021 | -0.028 | 0.052 | 0.074 |
|  | PM 2.5 | No lag | -.229^**^ | -.322^**^ | .162^**^ | .251^**^ | -.256^**^ | -.477^**^ | -0.015 | -0.011 | -.367^**^ | -.520^**^ |
|  |  | Lag 7 | -.135^*^ | -.192^*^ | .098^*^ | .163^**^ | -.363^**^ | -.520^**^ | 0.055 | 0.091 | -.383^**^ | -.550^**^ |
|  |  | Lag 14 | -0.042 | -0.046 | .095^*^ | .135^*^ | -.340^**^ | -.495^**^ | 0.105 | 0.157 | -.381^**^ | -.545^**^ |
|  | PM 10 | No lag | -.114^*^ | -.161^*^ | .216^**^ | .323^**^ | -.210^**^ | -.313^**^ | -0.050 | -0.071 | -.299^**^ | -.441^**^ |
|  |  | Lag 7 | -0.084 | -0.123 | .144^**^ | .231^**^ | -.312^**^ | -.454^**^ | -0.001 | -0.001 | -.326^**^ | -.480^**^ |
|  |  | Lag 14 | -0.044 | -0.068 | .100^*^ | .161^**^ | -.275^**^ | -.417^**^ | 0.032 | 0.045 | -.343^**^ | -.499^**^ |
